# Addressing barriers to accessing family planning services using mobile technology intervention among internally displaced persons in Abuja, Nigeria

**DOI:** 10.1016/j.xagr.2023.100250

**Published:** 2023-07-02

**Authors:** Sidney Sampson, Folake Oni, Oluwafisayo Ayodeji, Toluwani Oluwatola, Shiva Gab-deedam, Oluwatosin Adenipekun, Adebisi Adenipekun, Sunday Atobatele

**Affiliations:** Sydani Group, Abuja, Nigeria

**Keywords:** family planning, internally displaced people, mobile technology, reproductive health, underserved population

## Abstract

**BACKGROUND:**

Inadequate access to sexual and reproductive health services is prevalent among women of reproductive age in internally displaced people's camps. To address this, we implemented a mobile technology intervention, known as the Linking Underserved Populations to Sexual and Reproductive Health Services, in the Wassa Internally Displaced People's camp, Abuja, Nigeria.

**OBJECTIVE:**

This study aimed to assess the impact of the Linking Underserved Populations to Sexual and Reproductive Health Services Intervention in improving sexual and reproductive health services among women of reproductive age in Wassa Internally Displaced People's camp.

**STUDY DESIGN:**

A baseline survey was conducted among 105 women of reproductive age in the Wassa camp, followed by the deployment of the Linking Underserved Populations to Sexual and Reproductive Health Services intervention, which delivered family planning messages to camp residents between September 2020 and June 2021. This was followed by an endline survey. The FP utilization data in the camp health post were mined during the period of the intervention and were analyzed using Stata version 15 with a chi-square test performed at a significance level of 5%.

**RESULTS:**

Awareness of family planning among women of reproductive age in Wassa camp increased from 54.2% at baseline to 98% at endline. The major reason for refusal of family planning at baseline, which was a lack of spousal consent reduced from 29.5% to 7% at endline. Contraceptive prevalence rate increased from 18.1% at baseline to 26.2% at endline. In addition, 133 new family planning users were recorded at the endline. The uptake of family planning services recorded a strong association with family planning consultations (*P*<.05; χ2=6.41) and receipt of bulk short message service (*P*<.05; χ2=4.90).

**CONCLUSION:**

Mobile technology interventions are a recommended strategy that can increase family planning awareness and address barriers to family planning uptake.

**Glossary**
FP: Family planning
IEC: Information, education, and communication
IAWG: Inter-Agency Working Group
IDP: Internally displaced persons
FCT: Federal Capital Territory
LMICs: Low-and middle-income countries
LUPSS: Linking underserved populations to sexual and reproductive health services
NEMA: National Emergency Management Agency
NHMIS: National Health Management Information Systems
PPP: Pregnancy and postpartum
SEMA: State Emergency Management Agency
SMS: Short message service
SRH: Sexual and reproductive health
UNFPA: United Nations Population Fund
VFPP: Virtual family planning providers
WRA: Women of reproductive age

**AJOG Global Reports at a Glance**

**Why was this study conducted?**

This study aimed to assess the impact of the Linking Underserved Population to Sexual and Reproductive Health Services intervention in improving sexual and reproductive health services among women of reproductive age in Wassa Internally Displaced Persons’ camp in Abuja, Nigeria.

**Key findings**

Family planning (FP) awareness and contraceptive prevalence rate increased at the endline, whereas lack of spousal consent, which was the major reason for refusal of FP, reduced at endline.

There was a strong association between FP uptake and FP consultations/receipt of bulk short message service.

**What does this add to what is known?**

Lack of spousal consent had been a major barrier to the use of FP; however, this study showed that men should be involved in FP awareness and outreach to improve uptake of FP.

Mobile technology interventions could increase the uptake of FP services in low-middle-income countries.

### Introduction

Nigeria has experienced rising insecurity challenges in the past decade, including the Boko Haram insurgency in North East Nigeria, the farmers-herders crisis in the North Central and Southern states, and the activities of other armed nonstate actors.^1^ These challenges have resulted in the displacement of millions of people, who have either fled for their lives or lost their homes and livelihoods.^2^ Although seasonal flooding in different parts of the country has also caused some displacement,^3^ it is not comparable with the displacement caused by the insurgency, particularly the Boko Haram and the farmers-herders crisis.^2^

As a result of these conflicts, many displaced persons have become refugees in neighboring countries, whereas others have been forced to relocate within Nigeria and live in government-built, camp-like settlements, where they had to start their lives anew without their previous assets or means of livelihood.^4^ Currently, there are over 3.2 million internally displaced persons (IDPs) residing in various camps across different states of Nigeria, including the federal capital territory (FCT).^5^ The IDPs largely depend on support from government agencies such as the National Emergency Management Agency; State Emergency Management Agency donor agencies; humanitarian organizations such as the International Committee of the Red Cross, United Nations High Commissioner for Refugees, and the International Organization on Migration; and the host communities themselves.

Despite the presence of support agencies, IDP camps often lack access to essential services such as education, healthcare, nutritious meals, and adequate water and sanitation facilities.^6^^,^^7^ In addition, incidences of malaria, typhoid, and cholera outbreaks have been reported in some IDP camps.^8^^,^^9^ Owing to these numerous challenges, women in these IDP camps have been disproportionately affected because they have had to confront these difficulties alongside the limited availability of sexual and reproductive health (SRH) services.^8^^,^^10^

The United Nations Population Fund recognizes that access to basic SRH services including family planning, antenatal care, and delivery, is a fundamental right for women and girls, and it becomes even more critical for those living in IDP camps.^2^^,^^4^ The poor access to SRH services among the women living in these IDP camps has led to low uptake of FP services and worse maternal health outcomes, putting them at a higher risk of unwanted pregnancy, sexually transmitted infections, and maternal mortality.^11^^,^^12^ Although low uptake of FP services and high unmet need for FP are the challenges for policymakers in Nigeria as a whole, the situation is more dire among women living in IDP camps.^13^ Furthermore, the absence of national policies and guidelines on FP services in IDP camps has limited decision-making on who receives these services and under what circumstances, thereby resulting in suboptimal FP services in Nigeria.^13^^,^^14^

Regardless of policies and guidelines, additional challenges exist that limit the utilization of FP services in IDP camps. Challenges that have been documented in previous studies include cost, religious beliefs, spousal disapproval, cultural bias, fear of side effects, fertility desires, long distances and poor services of FP clinics, workload on healthcare workers leading to long waiting times at the clinic, limited knowledge and skills of providers, and inconvenience at the FP clinic.^15^, ^16^, ^17^, ^18^ Previous studies on FP in IDP situations show that published interventions for refugee communities have primarily focused on increasing access to care through supply-side improvements, as well as shifting behaviors and attitudes to enhance demand and use.^19^^,^^20^ The Inter-Agency Working Group in Reproductive Health in Crises’ 2012–2014 global review found that the scarcity of long-acting and permanent methods and other supply-side challenges, hinder accessibility to SRH services.^21^ The inadequacy of FP services in IDP camps has failed to measure up with the FP needs of the IDPs, thereby leading to a series of unmet needs with devastating consequences.

The disruption of essential health services during the COVID-19 pandemic further worsened access to FP in Nigeria,^22^ because lockdown measures imposed to curb the spread of the virus disrupted the supply chain and procurement of FP commodities. This resulted in a 30% to 50% drop in demand for FP services during the period of the lockdown.^23^ Although COVID-19 pandemic created health and economic challenges globally, it also increased opportunities for the adoption of digital health solutions among healthcare providers and users.^24^ The high mobile penetration in many low- and middle-income countries (LMICs) was utilized during the pandemic for various health programs and interventions such as health promotion, providing access to skilled-birth delivery, and monitoring treatment among others.^25^^,^^26^ Previous studies in Kenya have shown that mobile technology improves contraceptive use among women and couples.^27^ The study of Gbadegesin and Longe reported that IDPs in Nigeria have shown positive behavioral intention to use mobile health technologies.^28^

Given the opportunities and advantages that mobile technology offers, we implemented an intervention program tagged Linking Underserved Population to Sexual and Reproductive Health Services (LUPSS) in an IDP camp in the FCT between September 2020 and June 2021. This intervention utilized mobile technology to promote the uptake of FP services among women of reproductive age (WRA). The LUPSS intervention aimed to address FP challenges and improve the uptake of FP services among WRA in Wassa IDP camp. The objective of this study was, therefore, to assess the impact of the LUPSS intervention on improving SRH services among WRA in Wassa IDP camp, FCT Abuja, Nigeria.

### Materials and Methods

#### Study design

The study employed an action research design, and was conducted in Wassa IDP camp, southeast of the FCT Abuja, between September 2020 and June 2021.

#### Study location

Wassa IDP camp is located about 5 km from the city center and consists of 7 communities, with a population of 5121 IDPs. Most camp residents are IDPs from Adamawa, Borno, and Yobe states in North East Nigeria. Wassa IDP camp was purposively selected from the list of IDP camps in the FCT because of its population, and the presence of a government–owned health facility (health post) that provides SRH services. The health post, which was built in 2017 to serve the health needs of camp members, is served by 1 staff who is a community health worker.

#### Linking Underserved Population to Sexual and Reproductive Health Services intervention

The intervention implemented in Wassa IDP camp aimed to address barriers to FP uptake among WRA through 3 main components: demand side, supply side, and economic empowerment.

The demand-side intervention utilized mobile technology solutions, including a toll-free call service and biweekly, bulk, short message service (SMS). Trained registered nurses served as virtual family planning providers (VFPPs) and offered free FP consultations to camp residents through the toll-free call service. Camp residents were called weekly for 4 months using their contact details retrieved from existing camp records. The contact details were collated into a contact directory; most of the contacts on the directory were those of household heads. This was leveraged to tackle male spousal consent, which had been identified as one of the barriers to the uptake of FP. The VFPPs were equipped with a Google tracking sheet where they recorded phone call outcomes of consultations from camp residents that were called by the provider or called through the toll-free line. This was useful for the research team to observe how residents moved along the continuum of willingness to use FP to go to the facility to uptake the services. However, utilization data were collected from the FP National Health Management Information System Register (HMIS) at the health post.

Biweekly bulk SMS messages were sent to camp residents in both English and Hausa languages. These SMS messages aimed to provide factual information about FP and debunk popular myths and misconceptions about FP.

In addition, there were community sensitization activities through the implementation of mega and mini outreaches within the camp community. World Contraception Day was commemorated during the project with camp residents on September 26, 2020. On this day, over 1000 camp residents were in attendance for the outreach and were educated about FP through short discussions, context-specific information, education, and communication materials, and drama presentations that depicted the benefits of FP, as well as addressed common myths and misconceptions on FP. For project implementation and sustainability, community and religious leaders were decorated as FP champions during the outreach to ensure community ownership of demand creation for FP. These FP champions were trained and equipped with the FP contents and messages to lead mini outreaches which were to be implemented within the camp every 3 months after the intervention.

The supply-side component was a liaison with the FP unit of the FCT primary healthcare board to provide FP commodities utilization data from the health post in the camp as justification. The economic intervention component was the provision of microgrants of ($42) for 40 women in the IDP camp to cushion the effect of the COVID-19 pandemic on their livelihoods. This was done as an empowerment and to encourage the women but was not in any way factored into the study.

#### Sampling and study participants

The intervention lasted for 10 months (September 2020–June 2021), a baseline assessment was conducted in the camp at the commencement of the intervention, and an endline assessment was conducted after the intervention. For the baseline assessment, the minimum sample size of 105 was determined using Slovin formula at 90% confidence interval, and a 10% margin of error from the total population of 1359 WRA in the camp, which was obtained from the unofficial census count conducted by the Red Cross in the camp. The participants were selected using a multistage sampling technique. The camp was clustered into 7 communities that make up the camp. A total of 15 WRA were systematically selected from the houses in each cluster by selecting 1 WRA from even-numbered houses until the target was reached. For the endline, the same systematic approach was used to select the respondents, only that the odd-numbered houses were sampled. Of the 105 WRAs reached for the endline, only 103 WRA completed the survey.

##### Data tool

The data collection tool was developed by the project team, tested on a smaller population in another IDP camp, and validated by a national expert in the field of SRH in Nigeria. For the endline assessment, the data collection tool was modified to capture some details that emerged from the baseline assessment.

##### Data collection

Baseline and endline survey data were collected by trained data collectors using the data collection tool programmed on mobile devices. The FP utilization data in the camp from December 2019 to June 2021 was also mined from the facility HMIS register.

#### Outcome measures

Outcome measures of the study were to ascertain the: 1) additional users of FP services (number of additional WRA using a modern contraceptive method following the intervention); and 2) the contraceptive prevalence rate (modern methods) (the percentage of WRA who are using a modern contraceptive method following the intervention).

#### Statistical analysis

Sociodemographic data, self-reported awareness and utilization of FP data, and facility utilization data were collected. Descriptive statistics were obtained for sociodemographic variables, awareness, and uptake of FP services. A bivariate analysis was conducted to determine the association between the receipt of bulk SMS and virtual FP consultations on the uptake of FP. All analyses were conducted using Stata version 15, StataCorp LLC.

#### Ethical approval

The FCT Health Research Ethics approved the evaluation protocol and consent forms: FHREC/01/01/2007-31/08/2021. In addition to this, before the start of the surveys, all respondents were asked to consent to participate in the study. The consent forms contained a brief introduction to the research, clearly outlining the survey's benefits and risks and how personal identifiers from respondents will be kept confidential.

### Results

The baseline assessment was conducted among 105 WRA within the camp, whereas the endline assessment was conducted among 103 WRA. The details of the sociodemographic characteristics of the participant are presented in Table 1. The mean age for the women interviewed during the baseline assessment was 29.1±6.5 years, whereas the mean age for the endline survey was 28.3±6.6 years. Results showed that 32.4% and 45.6% of the respondents for the baseline and endline surveys, respectively, had no formal education. Details of the sociodemographics of survey respondents are presented in Table 1.

**Table 1:** Sociodemographic characteristics of survey respondents

Characteristic	Baseline assessment (n=105)	Endline survey (n=103)
Age (y)		
18–29 (%)	57.1	54.4
30–39 (%)	35.3	38.8
40–49 (%)	7.6	6.8
Religion		
Christian (%)	38.1	36.9
Muslim	61.9	63.1
Marital Status		
Single	1.0	2.9
Married	97.1	94.2
Separated	1.0	2.9
Widowed	1.0	0
Education		
None	32.4	45.6
Islamiyyat	20.0	11.7
Primary	24.8	29.1
Secondary	20.0	11.7
Post-secondary	2.9	1.9
Occupation		
Professional	1.0	1.0
Skilled manual	25.7	18.4
Unemployed	5.7	10.7
Unskilled manual	67.6	69.9

#### Baseline assessment

##### Awareness and utilization of family planning methods

The result of the baseline assessment showed that 54.2% of the respondents were aware of FP and FP methods. In terms of utilization of FP, results showed that most respondents (72%) were not using any form of FP whereas only about 18% were using modern FP methods. The remaining few participants (10%) were, however, using natural FP methods (withdrawal method and lactorrhea amenorrhea). The breakdown of the use of modern FP methods showed that 5% of the respondents used condoms, 42% used implants, 37% used injectables, and 16 % used pills (Figure 1).

**Figure 1:**
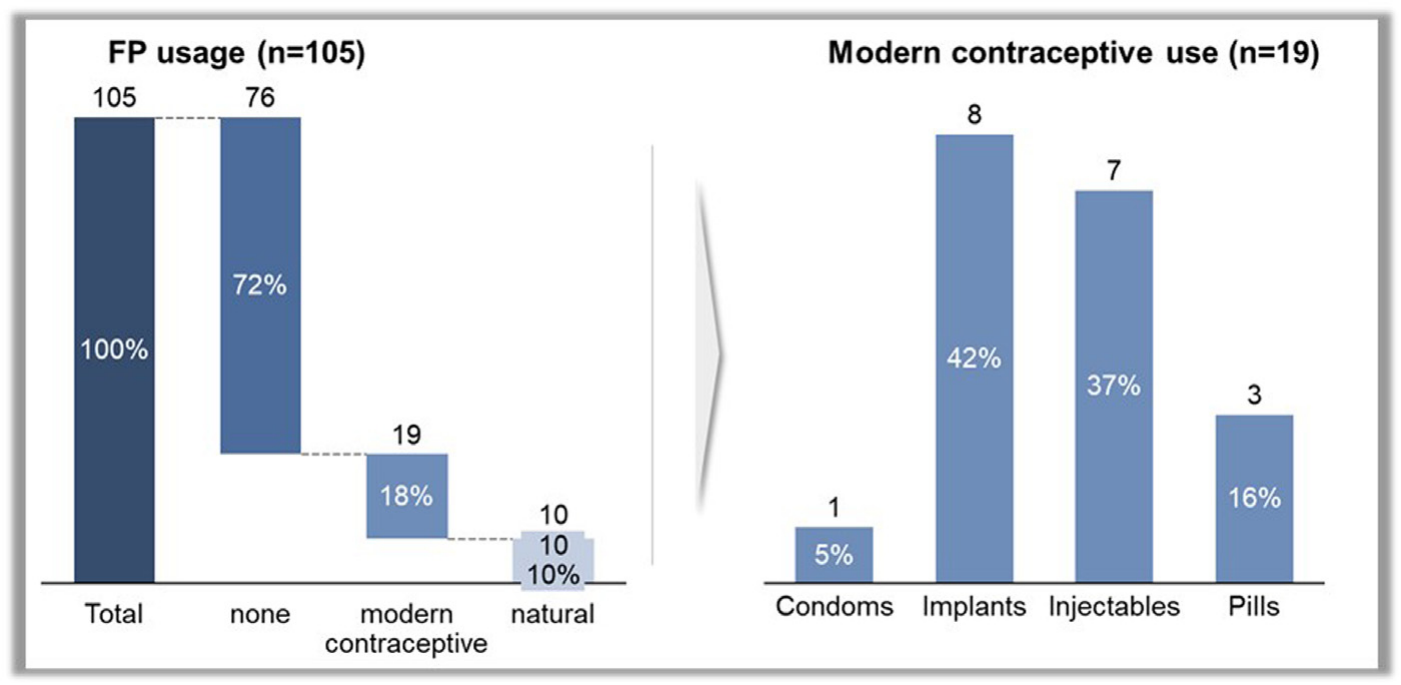
Baseline uptake of FP *FP*, family planning.

Sampson et al. Addressing barriers to accessing family planning services among internally displaced persons in Abuja, Nigeria. Am J Obstet Gynecol Glob Rep 2023.

##### Willingness to use family planning services

Out of the 76 participants that reported the nonusage of FP, a little over half (57.9%) were unwilling to use any method of FP whereas about 42.1% were willing to use FP method. About 8 reasons were highlighted as reasons for unwillingness to use any method of FP among the respondents at the baseline of the study, and lack of spousal consent ranked highest (29.5%), followed by pregnancy and postpartum (22.7%). Others are fear of adverse effects (18.2%), myths about FP (13.6%), the desire for more children (9.1%), sociocultural beliefs (2.3%), lack of funds (2.3%), and no reason (2.3%) Figure 2.

**Figure 2:**
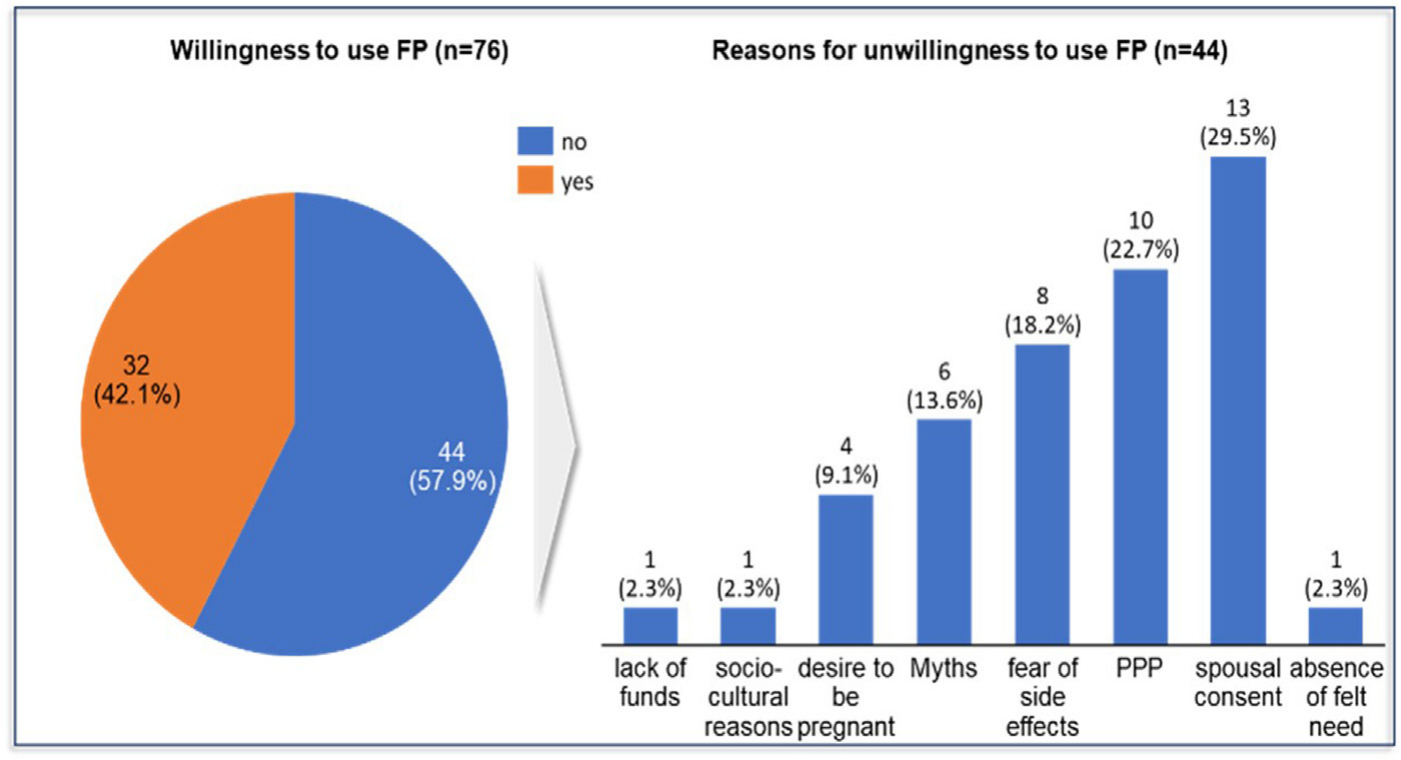
Baseline levels of willingness to use FP *FP*, family planning.

Sampson et al. Addressing barriers to accessing family planning services among internally displaced persons in Abuja, Nigeria. Am J Obstet Gynecol Glob Rep 2023.

#### Endline assessment findings

##### Awareness of family planning

Following the LUPSS intervention, there was an increase in the awareness of FP among WRA in Wassa IDP camp, from 54.2% at baseline to 98% at the endline. Results showed that about half of the respondents (46%) attributed their awareness of FP methods to LUPSS outreach implementation. Other respondents attributed awareness of FP methods to family and friends (24%), enlightenment at the health post (19%), radio (6%), and television (3%) programs as well as the LUPSS SMS or call (3%) (Figure 3).

**Figure 3:**
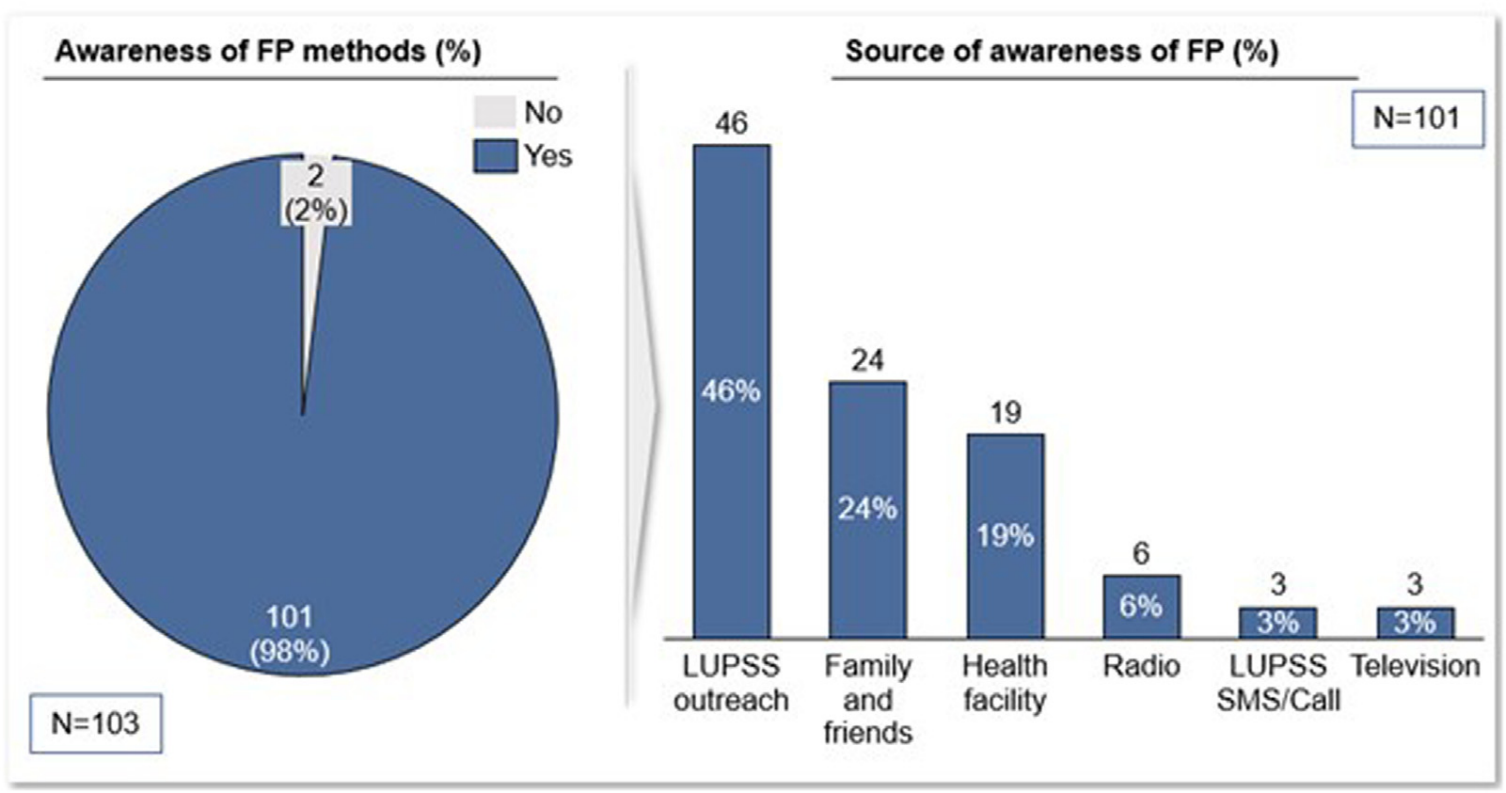
Awareness of FP *FP*, family planning.

Sampson et al. Addressing barriers to accessing family planning services among internally displaced persons in Abuja, Nigeria. Am J Obstet Gynecol Glob Rep 2023.

##### Family planning usage and desire to uptake family planning

At the endline, 72.3% of the respondents (n=73) were not using any method of family planning whereas 26.7% and 1.0% of the respondents were using modern and natural methods of FP, respectively (Figure 4). Of the 72.3% of respondents who were not using FP, 77% of them desired to use FP whereas the remaining 23% did not desire to use FP (Figure 5). Among respondents who were not using FP, the reasons included concerns related to pregnancy and postpartum (44%), desire to have more children (33%), fear of side effects (7%), spousal consent (7%), lack of knowledge (3%), and absence of felt need (7%) (Figure 6).

**Figure 4:**
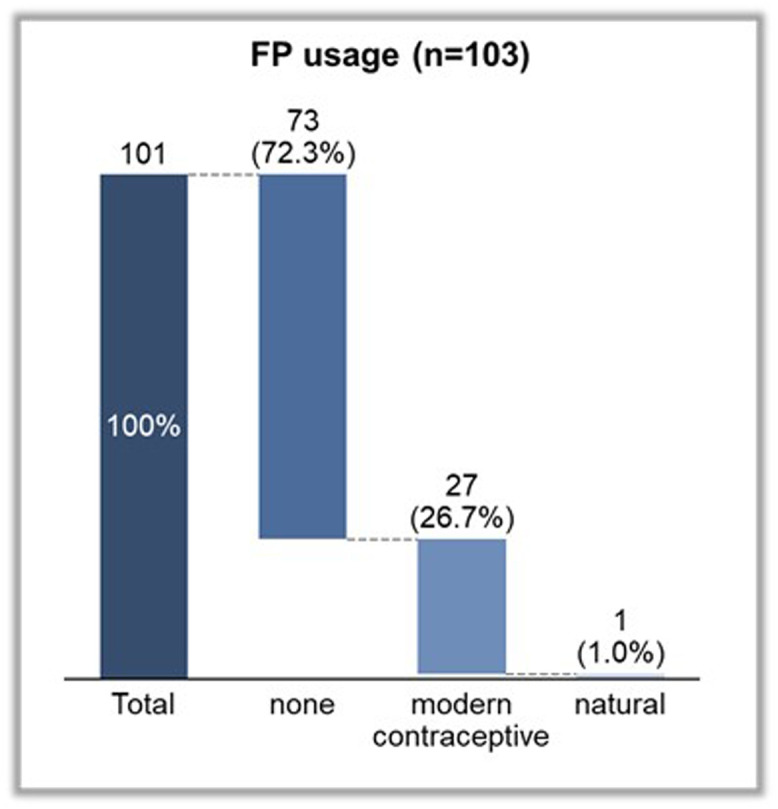
FP usage among WRA in Wassa IDP camp *FP*, family planning; *IDP*, internally displaced people's; *WRA*, women of reproductive age.

Sampson et al. Addressing barriers to accessing family planning services among internally displaced persons in Abuja, Nigeria. Am J Obstet Gynecol Glob Rep 2023.

**Figure 5:**
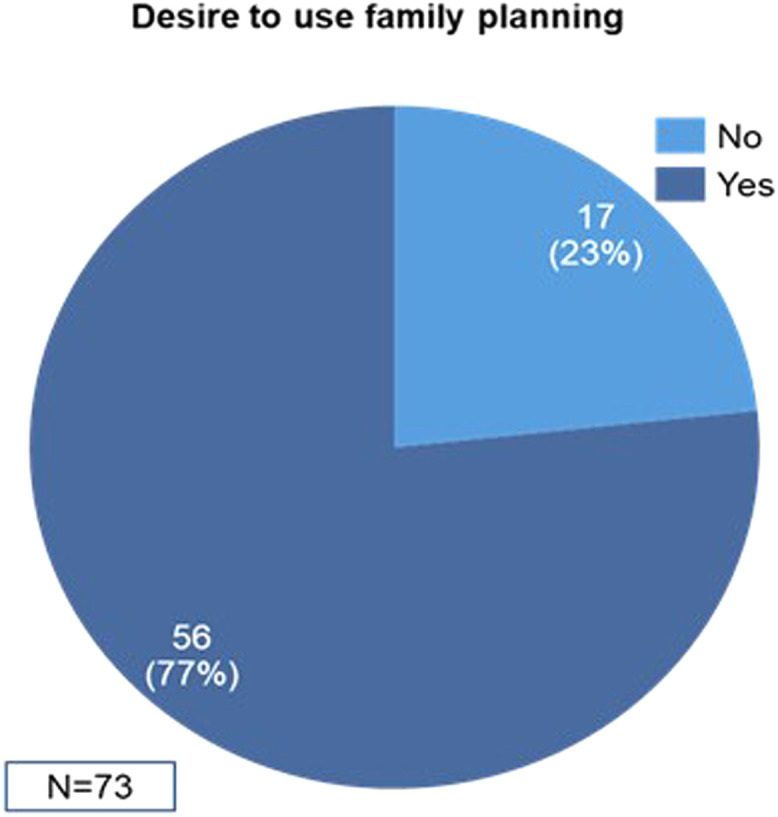
Desire to use FP among WRA in Wassa IDP camp *FP*, family planning; *IDP*, internally displaced people's; *WRA*, women of reproductive age.

Sampson et al. Addressing barriers to accessing family planning services among internally displaced persons in Abuja, Nigeria. Am J Obstet Gynecol Glob Rep 2023.

**Figure 6:**
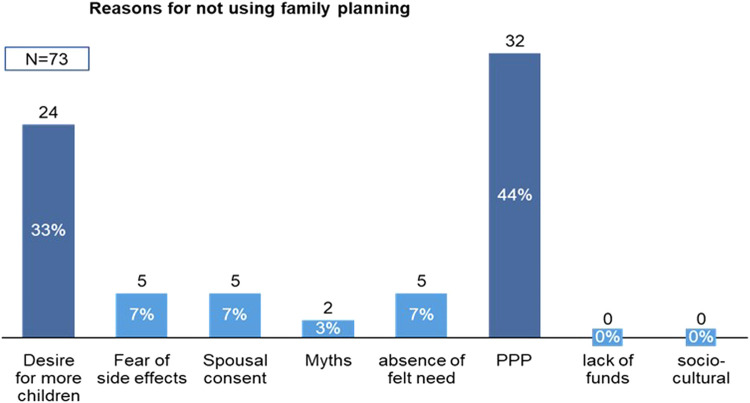
Reasons for not using FP among WRA in Wassa IDP camp *FP*, family planning; *IDP*, internally displaced people's; *WRA*, women of reproductive age.

Sampson et al. Addressing barriers to accessing family planning services among internally displaced persons in Abuja, Nigeria. Am J Obstet Gynecol Glob Rep 2023.

##### Uptake of family planning services

Utilization data at the camp clinic showed that there were 133 new FP users during the intervention period (Table 2). Trend analysis of new users showed that the number of new users peaked during the month of the mega outreach which was conducted to commemorate world contraceptive day. Results of the data from the camp's health post also showed a 54% increase in FP users at the endline of the intervention. The result of the trend analysis of the total number of FP users at the Wassa IDP camp health post showed a steady increase throughout the period of intervention, specifically from 244 FP users at the baseline to 377 FP users at the endline (Figure 7). Results also showed that the contraceptive prevalence rate increased from 18.1% at baseline to 26.2% at the endline (Table 2).

**Table 2:** Summary of outcome variables

Outcome	Baseline	Endline
Additional users of contraceptives	—	133
Contraceptive prevalence rate (modern methods)	18.1%	26.2%

**Figure 7:**
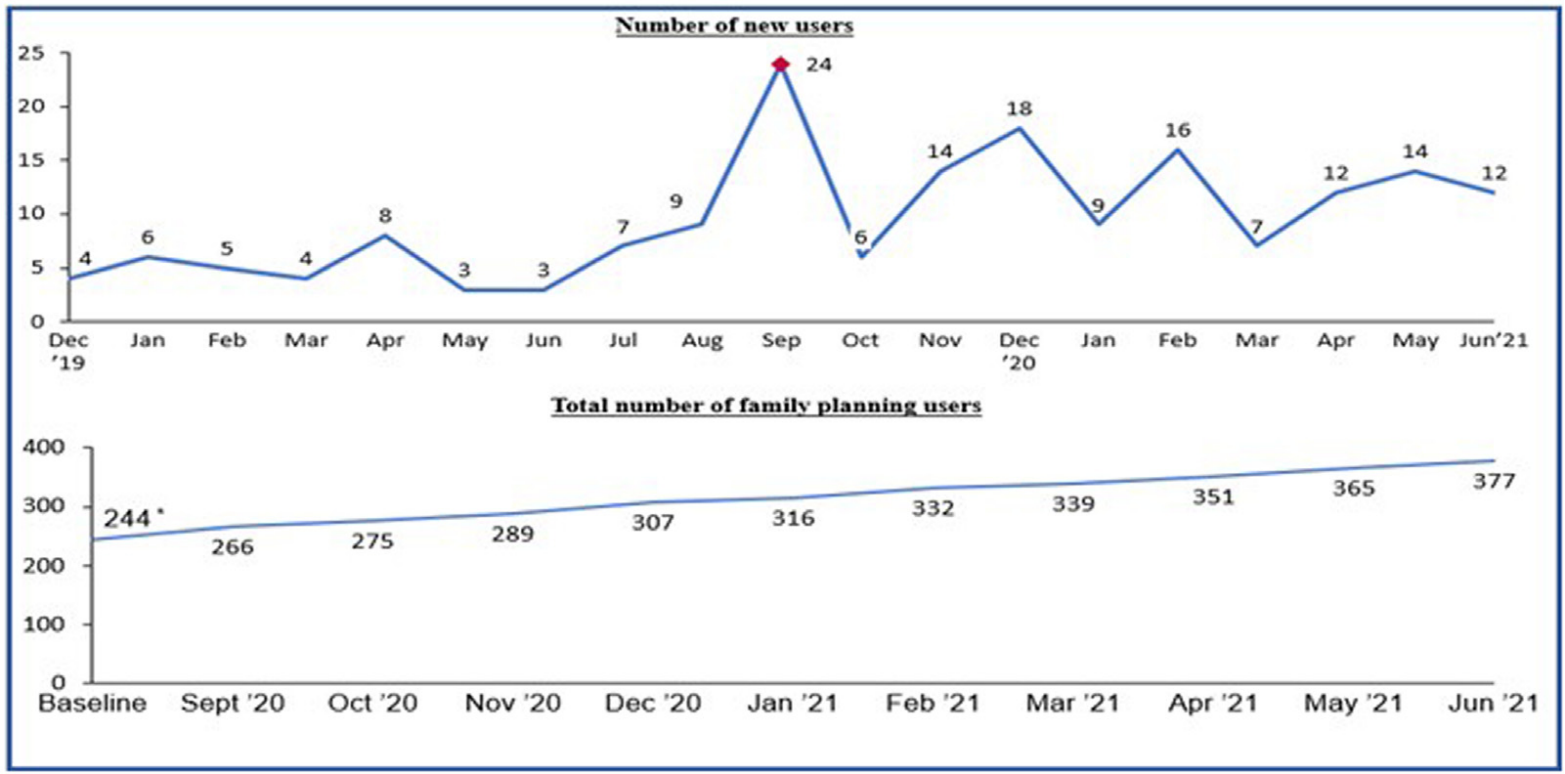
Trend analysis of FP users in Wassa IDP camp *FP*, family planning; *IDP*, internally displaced people.

Sampson et al. Addressing barriers to accessing family planning services among internally displaced persons in Abuja, Nigeria. Am J Obstet Gynecol Glob Rep 2023.

##### Influence of mobile technology on the uptake of family planning

The result of the bivariate analysis showed a strong association between the call toll-free line that was used for FP consultations and the uptake of FP services (*P*<.05; χ2=6.41). In the same vein, there was a strong association between the receipt of bulk SMS on FP services and the uptake of FP services (*P*<.05, χ2=4.90) (Table 3).

**Table 3:** Utilization of FP services by intervention received (n=103)

		Did not use FP	Used FP		
Response	Total number (%)	Number (%)	Number (%)	*P*-value	χ2
Virtual family consultation (call on toll-free line)					
No	42 (40.8)	32 (31.1)	10 (9.7)	.011	6.419
Yes	61 (59.2)	23 (22.3)	38 (36.9)		
Receipt of bulk SMS					
No	47 (45.6)	29 (28.2)	18 (17.5)	.027	4.906
Yes	56 (54.4)	12 (11.6)	44 (42.7)		

*FP*, family planning.

### Discussion

This study assessed the impact of the LUPSS intervention in improving FP usage among WRA in Wassa IDP camp, FCT Abuja. The LUPSS project which was designed to demonstrate a proof of concept for a mobile technology intervention to improve the uptake of FP services among WRA in IDP camps has further shown some barriers and challenges that are associated with the uptake of FP services, among the women in these camps and by extension, validated what has been reported in existing literature.^13^^,^^29^^,^^30^ Findings from the study highlighted a generally low uptake of FP services because <1 in 4 of the WRA in Wassa IDP camp are using any FP method, at both baseline and endline of the LUPSS intervention. This is similar to the study of Idris et al, which highlighted the challenges of scaling up FP services that are being faced by reproductive health service providers in Nigeria.^31^ Although this study showed low uptake of FP services, it highlighted the reasons behind the low uptake in low-income countries like Nigeria.

At baseline, our study showed that the lack of FP awareness limited the uptake of FP services, and this was in tandem with other studies.^13^^,^^32^^,^^33^ Furthermore, just a little over half of the respondents showed a willingness to use FP services at the baseline, and this was primarily owing to lack of spousal consent, which had reportedly been a major barrier to the use of FP.^34^, ^35^, ^36^ The lack of spousal consent however reduced significantly at the endline and could be attributed to the LUPSS intervention. The LUPSS intervention has shown that targeted awareness and outreach that includes the men could play a huge role in reducing the lack of spousal consent as a barrier to the uptake of FP. This is because in the African culture, especially in Nigeria, men play a significant role in families and are the major decision makers. As part of the LUPSS intervention, the importance of fostering communication and collaboration between spouses, especially on health-related decisions was emphasized, thereby helping to resolve difficulties in the usage of FP services.^29^^,^^35^ It is also important to introduce and incorporate more male-friendly options into FP services.^36^

Other reasons identified at baseline such as myths about FP, fear of side effects, lack of funds, and sociocultural concerns, which were contributory barriers to the uptake of FP services, align with studies that have found these factors, among others, to contribute to the suboptimal uptake of FP services among Nigerian women^13^^,^^32^ These other reasons, however, reduced significantly at endline, and this could be attributed to the LUPSS intervention. Given the young average age of the WRA in this study, it is understandable that the primary reason for not using FP at both baseline and endline was the desire to have more children and concerns related to pregnancy and postpartum.^13^^,^^36^^,^^37^

Before the LUPSS intervention, there was low uptake of modern contraceptives and a high unmet need for family planning among WRA in the camp with the results from the baseline and endline survey showing that uptake of modern FP methods is below the average contraception rate in the FCT.^30^ This means that more intervention and awareness programs are needed in the WASSA IDP camp and other IDP camps to bridge this gap. The uptake of modern FP methods, however, significantly improved and steadily increased throughout the period of the intervention, and this could be attributed to the LUPSS intervention.

There was an association between the uptake of FP services and the mobile technology intervention that was deployed in this project, the virtual family planning consultations (VFPC) and bulk SMS. This suggests that continuous FP consultations and SMS-bearing FP messages could increase the uptake of FP services in Wassa IDP camp, and by extension, other IDPs. This can also be applied to FP programs or interventions to increase or improve FP uptake. The design of the VFPC and bulk SMS to target household heads, which was done to improve spousal consent for FP services aligns with recommendations of studies that have suggested male involvement in FP services to boost uptake.^38^^,^^39^ The design of the project to engage religious and community leaders, especially in Northern Nigeria, has been an effective strategy for improving the uptake of modern contraceptive services in Nigeria.^40^ All of these contributed to the success of the LUPSS project. The overall outcome of these findings aligns with previous studies that evaluated the impact of mobile technology interventions on the uptake of FP services.^41^

#### Study strengths and limitations

The LUPSS intervention has some strengths and limitations. One major strength was the active engagement of stakeholders in Wassa IDP camp, including religious and traditional leaders, who served as FP champions. This approach ensured project ownership and sustainability. In addition, the intervention utilized the existing government–owned health facility in Wassa IDP camp, allowing camp members to interact with a familiar healthcare facility, rather than introducing a new one that would require time for integration. This ensured continued access to FP services beyond the intervention's duration because the camp's health facility would remain operational.

However, a limitation of the study was that the objective of the study was not to compare the baseline and endline of the intervention directly. Instead, the aim was to assess the impact of this intervention on the camp population. Consequently, different respondents were assessed at the baseline and endline stages. Although this approach may have influenced the findings, it was adopted to minimize Hawthorne effect, where study participants may change or improve their behavior because they are being studied.

### Conclusion

The study conducted in the Wassa IDP camp, FCT Abuja, revealed important findings regarding FP awareness and utilization among WRA. At the baseline, WRA in the camp had a low level of FP awareness, but this increased significantly by the endline. The study also identified that the primary reasons for nonusage of FP among WRA were the desire to have more children and concerns related to pregnancy and postpartum. Notably, the study showed that targeted interventions aimed at engaging males resulted in a reduction of the prominent barrier of spousal consent for FP usage. In conclusion, the LUPSS intervention, which employed mobile technology solutions, played a crucial role in increasing FP awareness and addressing barriers to FP uptake among WRA in the Wassa IDP camp. The findings highlight the effectiveness of the intervention in promoting FP services and improving SRH outcomes in the camp population.
